# Pleuropulmonary blastoma manifesting as spontaneous pneumothorax: an unusual presentation

**DOI:** 10.1590/0100-3984.2017.0189

**Published:** 2019

**Authors:** Isa Félix Adôrno, Rômulo Florêncio Tristão Santos, Bernardo Bacelar de Faria, Edson Marchiori, Thiago Franchi Nunes

**Affiliations:** 1 Hospital Universitário Maria Aparecida Pedrossian da Universidade Federal de Mato Grosso do Sul (HUMAP-UFMS), Campo Grande, MS, Brazil.; 2 Screenlab, Campo Grande, MS, Brazil.; 3 Universidade Federal do Rio de Janeiro (UFRJ), Rio de Janeiro, RJ, Brazil.

**Figure 1:**
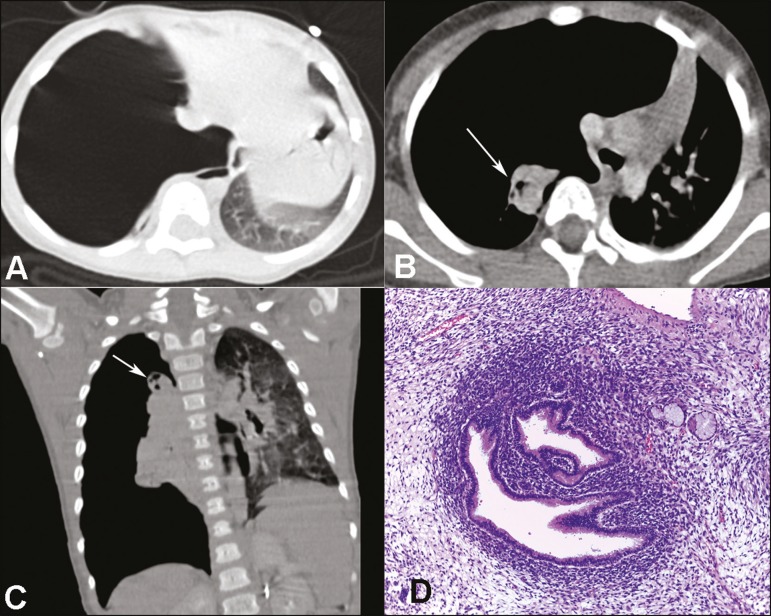
CT scan of the chest showing a voluminous pneumothorax on the right (**A**), extensive cavitation (arrows), and atelectasis of the right lung (**B,C**). **D:** Histological section showing the biphasic component of the neoplasm: the solid area with a dense immature component around the normal pulmonary epithelium (cambium layer); and the adjacent spindle-cell component in which there were typical cytoplasmic striations (not shown). Hematoxylin-eosin staining (magnification, ×200).

Dear Editor,

A previously healthy two-year-old female patient presented to the emergency department with sudden-onset dyspnea and right-sided chest pain. Physical examination revealed tachypnea, absence of breath sounds on the right and distention of the ipsilateral jugular vein. Routine laboratory tests showed no abnormalities. A chest X-ray showed hypertensive pneumothorax on the right. A computed tomography (CT) scan of the chest revealed, in addition to the voluminous pneumothorax, extensive cavitation and atelectasis in the right lung (Figures 1A, 1B, and 1C). We opted for thoracic surgery involving immediate drainage of the pneumothorax and, during the same hospitalization, resection of the pulmonary lesion. The macroscopic pathological examination revealed a circumscribed, subpleural, nodular lesion that was solid-cystic and friable, measuring 1.4 cm in diameter, together with pleural rupture. Histologically, we also observed a neoplasm with foliaceous and solid patterns, together with cystic areas, lined with normal respiratory epithelium (Figure 1D). The neoplastic cells presented two predominant patterns: an undifferentiated immature (blastomatous) component, mainly in the subepithelial region; and a spindle cell component with rhabdomyoblastic differentiation, comprising a few anaplastic cells and numerous atypical mitoses. We observed no chondral differentiation or necrosis. The final diagnosis was type II pleuropulmonary blastoma (PPB). The subsequent staging did not reveal any metastatic dissemination of the disease. After surgery, the patient recovered quickly, with no respiratory symptoms. A follow-up chest X-ray showed full expansion of the affected lung. There was no need for postoperative radiotherapy.

PPB is an aggressive intrathoracic malignant neoplasm that mainly affects children under five years of age and, although rare, is the most common primary malignant neoplasm of the lung in childhood^(^^1^^)^. It derives from primitive embryonic cells that arise during the development of the lung, similar to what is observed in other childhood neoplasms. These primitive cells are associated with other congenital pulmonary cystic malformations, some of which evolve to an aggressive neoplasm, with possible sarcomatous transformation, demonstrating the potential of multidirectional differentiation of stem cells^(^^2^^)^. In general, PPB manifests as an intrapulmonary subpleural mass and is characterized histologically by primitive blastomatous and sarcomatous differentiation containing non-neoplastic pulmonary epithelial elements.

With the development of new technologies, imaging studies are becoming increasingly more important in pediatrics^(^^3^^-^^7^^)^. However, there have been few articles describing the imaging findings of PPB^(^^8^^)^. Pneumothorax is a common presentation in type I (purely cystic) PPB and type II (mixed) PPB^(^^9^^,^^10^^)^. PPB is highly aggressive in its type III (solid) form, with recurrence and metastases. The most common metastases are those to the lung/pleura, central nervous system, and musculoskeletal system. Although CT of the chest is the most widely used technique for investigating a pulmonary mass, magnetic resonance imaging is useful because it can better demonstrate the origin of the mass, its anatomical relationships, and the involvement of adjacent structures. Radiographic findings, in general, are not sufficient for a definitive diagnosis, lung biopsy being essential for the final diagnosis.
